# Dietary Isoflavones Intake and Gastric Cancer

**DOI:** 10.3390/nu16162771

**Published:** 2024-08-20

**Authors:** Arianna Natale, Federica Fiori, Maria Parpinel, Claudio Pelucchi, Eva Negri, Carlo La Vecchia, Marta Rossi

**Affiliations:** 1 Department of Clinical Sciences and Community Health, Dipartimento di Eccellenza 2023–2027, University of Milan, 20133 Milan, Italy; arianna.natale@unimi.it (A.N.); marta.rossi@unimi.it (M.R.); 2 Department of Medicine-DAME, University of Udine, 33100 Udine, Italy; federica.fiori@uniud.it (F.F.); maria.parpinel@uniud.it (M.P.); 3 Department of Medical and Surgical Sciences, University of Bologna, 40126 Bologna, Italy; eva.negri@unibo.it

**Keywords:** isoflavones, gastric cancer, flavonoids, diet, risk, legumes

## Abstract

Dietary isoflavones have been associated with a lower risk of gastric cancer (GC), but the evidence for this association is still limited. We investigated the association between isoflavone intake and GC risk using data from a case–control study including 230 incident, histologically confirmed GC cases and 547 controls with acute, non-neoplastic conditions. Dietary information was collected through a validated food frequency questionnaire (FFQ) and isoflavone intake was estimated using ad hoc databases. We estimated the odds ratios (OR) and the corresponding 95% confidence intervals (CI) of GC using logistic regression models, including terms for total energy intake and other major confounders. The OR for the highest versus the lowest tertile of intake was 0.65 (95%CI = 0.44–0.97, *p* for trend = 0.04) for daidzein, 0.75 (95%CI = 0.54–1.11, *p* for trend = 0.15) for genistein, and 0.66 (95%CI = 0.45–0.99, *p* for trend = 0.05) for total isoflavones. Stratified analyses by sex, age, education, and smoking showed no heterogeneity. These findings indicate a favorable effect of dietary isoflavones on GC.

## 1. Introduction

Gastric cancer (GC) is the fifth cause of death from cancer globally and also remains a frequent. cancer in Italy [1]. Although GC mostly affects countries with low or middle-income economies, particularly in Asia, this malignancy caused approximately 50,000 deaths in Europe in 2022 and will cause over 8200 deaths in Italy in 2024 [1,2,3]. Infection with *Helicobacter pylori*, tobacco smoking, heavy alcohol consumption and selected dietary habits are strongly associated with the risk of GC. In particular, high consumption of red meats and salt-preserved foods, and low consumption of fruit have been associated with excess GC risk [4,5,6].

Flavonoids are chemical compounds found in plant food and are among the candidates explaining the favorable effect of plant-based food on GC risk [4,7]. Isoflavones, represented by genistein and daidzein, are a class of flavonoids characterized by a peculiar estrogen-like structure, and are mainly, but not exclusively, found in legumes [8]. Various biological mechanisms mediate the effect of isoflavones on GC, including their antioxidant and growth inhibitor activities [9]. Genistein lowered GC cell stem-like properties by downregulating Gli1 and CD44 expression [10], as well as other stem cell markers including OCT-4, Sox2 and Nanog [11]. Additionally, by suppressing COX-2 [12] and upregulating the tumor suppressor PTEN [13], it inhibited proliferation of GC cells. In nude mice, genistein decreased the Bcl-2/Bax ratio, inducing apoptosis in transplanted human GC cells [14], and, by inhibiting the same pathway, daidzein caused the apoptosis of human GC cells [15]. Daidzein, genistein and isoflavones’ aglycones induced cytostasis in transplanted human GC cells and a reduction in cachexia in mice [16]. Among them, aglycones exerted the most effective antitumoral action. Moreover, in human gastric cells, equol inhibits growth and proliferation and induces apoptosis [17,18] by enhancing the ERK1/2 pathway and dephosphorylating PAkt at Thr450. Daidzein also undergoes a two-step transformation by gut microbiota, resulting in dihydrodaidzein (DHD), O-desmethylangolensin (O-DMA) and then equol [19,20,21], which are bioactive compounds with a strong radical-scavenger activity [19,22].

A meta-analysis reported a pooled relative risk of 0.89 (95%CI = 0.77–1.03) for the highest versus the lowest levels of isoflavone intake from cohort studies, as well as an OR of 0.99 (95%CI = 0.72–1.36) from case–control studies [23].

We estimated the content of isoflavones from the European database “Vegetal Estrogens in Nutrition and the Skeleton” (VENUS) [24] on data from an Italian case–control study in order to investigate the relationship between dietary isoflavones and GC risk.

## 2. Materials and Methods

We used data from a case–control study on GC conducted in the greater Milan area, Italy, between 1997 and 2007. The cases featured 230 individuals, 143 males and 87 females, aged 22–80 years (median age 63 years) with incident, histologically confirmed GC who were admitted to general and major teaching hospitals. The controls were 547 individuals, 286 males and 261 females, aged 22–80 years (median age 63 years) with no history of cancer. They were enrolled in the same hospitals as cases for non-neoplastic acute conditions that were unrelated to risk factors for GC or to long-term modification of diet. Controls were matched to cases by sex and age with a ratio of 2:1 for males and 3:1 for females. Twenty percent of controls were admitted for traumatic disorders, sprains and fractures; 23% were admitted for other orthopedic conditions; 22% were admitted for acute surgical conditions, and 35% were admitted for other miscellaneous illnesses. Less than 5% of the contacted subjects refused to participate.

The participants were interviewed during their hospital stay by trained interviewers using a structured questionnaire, which included questions about personal and socio-demographic characteristics and lifestyle habits, such as tobacco and alcohol consumption, personal medical history and family history of GC in first degree relatives. Individuals who had quit smoking for at least one year were considered to be former smokers.

Dietary habits were assessed through a food frequency questionnaire (FFQ) which was satisfactorily tested for reproducibility [25] and validity [26]. The FFQ included questions on the weekly consumption of 78 foods items, recipes, or food groups, including beverages, in the 2 years preceding the diagnosis (for cases) or the hospital admission (for controls). Intakes lower than once a week but equal or higher than once per month were coded as 0.5. In each section, open questions were used to assess the frequency of consumption and the portions of food items that were not included in the FFQ (one open question each for milk and hot beverages, cereal products, meat and other first courses and desserts; two open questions each for side dishes and fruit).

Daily energy intake was estimated using an Italian food composition database [27,28], and data on isoflavone intake were derived from the VENUS database [24] (accessed on 8 May 2008) and other sources when needed [29,30,31].

We derived the odds ratios (ORs) of GC and the corresponding 95% confidence intervals (CIs) according to tertiles (on the distribution of controls) of isoflavone intake using logistic regression models. The models included terms for sex, age (quinquennia), years of education (<7, 7–11, ≥12), year of interview, tobacco smoking status (never, former, current <15 and ≥15 cigarettes per day), and total energy intake (tertiles). We also adjusted for vegetable and fruit consumption. In addition, we analyzed the ORs of GC by the strata of sex, age, education and smoking, and we evaluated the heterogeneity by the likelihood ratio test.

We performed all analyses with SAS software version 9.4 (SAS Institute, Inc., Cary, NC, USA).

## 3. Results

Table 1 shows the distribution of 230 cases of GC and 547 controls according to selected factors. Cases tended to be less educated, were more frequently current or former smokers, and had a higher GC family history than controls.

Table 2 gives the mean daily intake of daidzein, genistein and total isoflavones among cases and controls, as well as the ORs of GC according to tertiles of intake. The mean intake was 21.8 μg/day for daidzein, 24.4 μg/day for genistein and 46.2 μg/day for total isoflavones. Comparing the third versus the first tertile, the OR was 0.65 (95%CI = 0.44–0.97, *p* for trend = 0.04) for daidzein, 0.75 (95%CI = 0.54–1.11, *p* for trend = 0.15) for genistein and 0.66 (95%CI = 0.45–0.99, *p* for trend = 0.05) for total isoflavones.

**Figure 1:**
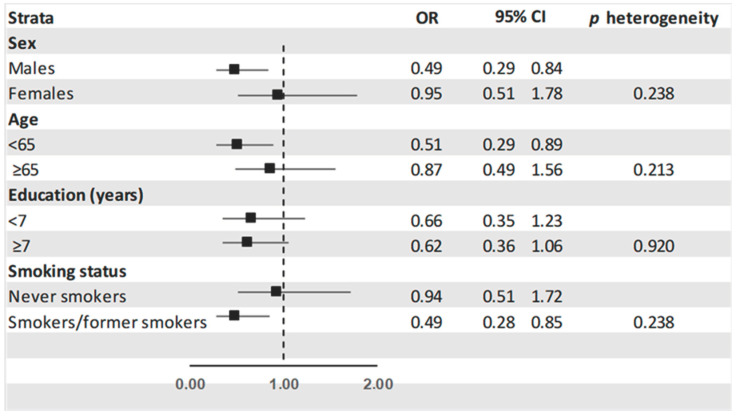
Odds ratios ^a^ (OR) of gastric cancer and corresponding 95% confidence intervals (C) for the highest versus the lowest tertile of isoflavone intake in the strata of selected characteristics. Italy 1997–2007. **^a^** Derived from the logistic regression model adjusting for sex, age, education, year of interview, smoking status, and total energy intake.

Figure 1 shows the ORs and the corresponding 95%CI of GC for the highest versus the lowest tertile of total isoflavone intake in the strata of sex, age, education, and smoking status. No heterogeneity trends was observed across strata.

## 4. Discussion

Our study indicates an inverse association between dietary isoflavones and GC risk. The OR estimates were consistent across strata of major covariates.

The anticarcinogenic effect of isoflavones has been assessed in vitro and in vivo [10,11,12,13,14,15,16,17,18,19,22]. Besides the radical-scavenging and antiproliferative mechanisms [9], the favorable effect on GC can be linked to the interplay between isoflavones and gut microbiota. In GC patients, the short chain fatty acid (SCFA) production is decreased [32]. In vivo models and in the Simulator of the Human Intestinal Microbial Ecosystem, isoflavones enhanced the SCFA production [33,34]. Equol levels also have been related to the presence of the two SCFAs butyrate and propionate [35]. This indicates that dietary isoflavones might influence GC risk through a favorable effect on SCFA production, with their effects potentially being enhanced by a healthy gut microbiota. Moreover, in vitro, genistein inhibited the proliferation of *H. pylori*, a major GC risk factor [36].

Isoflavone intakes have been inversely related to GC risk, but the association is still controversial [23,37]. Various studies on this subject were conducted in Asia [23], where the average dietary isoflavones intake is about 10 times greater compared to Europe [38,39] due to their high soy product consumption, and other studies came from European countries [38,40], where food sources of isoflavones are more varied [41,42]. In our population, the isoflavones are mainly derived from non-soy legumes, cooked vegetables, and fruits, allowing us to evaluate the effect of non-soy dietary isoflavones on GC.

Our results were favorable despite a modest mean isoflavone intake in our population. This can be related to various aspects influencing isoflavone bioavailability. Firstly, it can be affected by the interaction with the intake of other dietary factors, such as fermentable fiber, which are positively related to a greater equol production [43] and to an improved bioavailability [44,45]. Increased equol production is also directly related to a high PUFA–SFA ratio and a vitamin A-rich diet [46]. Secondly, bioavailability may be influenced by cooking methods and food characteristics [46,47]. High processing temperatures have been demonstrated to enhance isoflavones bioavailability [47,48], and isoflavone aglycones, which are more bioavailable than glycosides [49], are often found in thermally processed foods [47,50,51]. Moreover, in our population, consuming boiled or canned non-soy legumes may have provided beneficial amounts of aglycones. The bioavailability of isoflavones may also vary according to sex, age, ethnicity (e.g., equol producers are lower in Caucasian than in Asian populations), dietary habits and health status [52,53]. However, we adjusted for some of these covariates. Isoflavones may be also considered a proxy for fruit and vegetable consumption [54] and other favorable aspects (e.g., Mediterranean diet [55]), but when we adjusted for vegetable and fruits, our estimates changed only marginally.

In our study, the inverse association tended to be more pronounced for daidzein than genistein. The mechanisms by which daidzein and genistein may act on GC are diverse, but there is no clear explanation for this. The isoflavones’ estrogenic-like activity should be considered in GC risk modulation, as exposure to estrogens has been associated with a lower GC risk in both males [56] and females [57], and the outcome may be related to the magnitude of the estrogenic effect [58]. Exposure to tobacco may also affect the isoflavones’ effect on GC risk. Lower levels of miR-218 have been associated with the activation of the cancer-promoting transcription factor NF-kB and have been observed in GC cells and the bronchial epithelium of smokers [59]. The inhibitory effect of genistein on NF-kB [12] may vary between smokers and non-smokers, though the issue remains unsettled.

A limitation of this study was the unavailability of data about *H. pylori* infection. However, case–control studies have limited ability to test for *H. pylori*, as the markers of infection fall after the onset of GC [60,61]. Regarding selection bias, cases and controls were recruited in comparable hospital settings, and the response rate was nearly complete. All controls admitted for conditions linked to long-term dietary changes, or with chronic pathologies, were excluded. To minimize information bias, cases and controls were interviewed by the same trained interviewers in similar settings using a satisfactorily validated and reproducible FFQ [25,26], although this was not specifically designed to evaluate isoflavone intake. As a strength, the use of a European food composition database [24] allowed us to improve the estimate of isoflavone intake in our Italian population. Additionally, we were able to account for many potential confounding variables, including energy intake. A major strength of this analysis was to provide findings from an Italian population, where isoflavone intake derives from multiple food sources and information on the role of isoflavones in regard to GC risk is still scant.

## 5. Conclusions

Isoflavones, mainly derived from non-soy legumes, appear to exert a favourable impact on gastric cancer risk in an Italian population. The trends in risk were significant for total isoflavones and daidzein. This supports the message that a diet rich in legumes has a protective effect on gastric carcinogenesis.

## Figures and Tables

**Table 1:** Distribution of 230 cases of gastric cancer and 547 controls according to sex, age, education, smoking status and family history of gastric cancer. Italy, 1997–2007.

Characteristics	Cases		Controls	
	No.	(%)	No.	(%)
Sex				
Males	143	62.2	286	52.3
Females	87	37.8	261	47.7
Age				
<50	39	17	97	17.7
50–60	58	25.2	137	25.1
60–70	86	37.4	202	36.9
≥70	47	20.4	111	20.3
Education (years)				
<7	95	41.8	236	43.5
7–11	86	37.9	174	32
≥12	46	20.3	133	24.5
Smoking status				
Never smokers	96	41.9	261	47.8
Former smokers	75	32.8	167	30.6
Current smokers				
<15 cigarettes/day	25	10.9	49	9
≥15 cigarettes/day	33	14.4	69	12.6
Family history of gastric cancer				
No	200	87.0	516	94.3
Yes	30	13.0	31	5.7

**Table 2:** Odds ratios ^a^ (OR) and 95% confidence intervals (C) of gastric cancer for daidzein, genistein and total isoflavone tertiles of intake among 230 cases and 547 controls. Italy, 1997–2007.

	Mean (SD) ^b^	Tertiles			*p* for Trend
		I	II	III	
Daidzein (μg/day)					
Cut-off	21.8 (12.8)	-	15.7	24.4	
Controls:cases		182:88	182:72	183:70	
OR (95%CI)		1	0.71 (0.48–1.05)	0.65 (0.44–0.97)	0.04
Genistein (μg/day)					
Cut-off	24.4 (14.2)	-	17.5	26.6	
Controls:cases		183:83	181:73	183:74	
OR (95%CI)		1	0.81 (0.55–1.20)	0.75 (0.54–1.11)	0.15
Total isoflavones (μg/day)					
Cut-off	46.2 (23.2)	-	35.3	51.5	
Controls:cases		182:87	183:73	182:70	
OR (95%CI)		1	0.74 (0.50–1.09)	0.66 (0.45–0.99)	0.05

^a^ Derived from logistic regression model adjusting for sex, age, education, year of interview, smoking, and total energy intake. ^b^ Defined among controls.

## Data Availability

The data presented in this study are available upon justified request from the corresponding author.
